# Follow-up care for premature children: the repercussions of the COVID-19 pandemic*

**DOI:** 10.1590/1518-8345.4759.3414

**Published:** 2021-04-12

**Authors:** Rosane Meire Munhak da Silva, Letícia Pancieri, Adriana Zilly, Fabiana Aparecida Spohr, Luciana Mara Monti Fonseca, Débora Falleiros de Mello

**Affiliations:** 1 Universidade Estadual do Oeste do Paraná, Foz do Iguaçu, PR, Brazil.; 2 Universidade de São Paulo, Escola de Enfermagem de Ribeirão Preto, PAHO/WHO Collaborating Centre for Nursing Research Development, Ribeirão Preto, SP, Brazil.; 3 Hospital Ministro Costa Cavalcanti, Centro de Atendimento à Gestante, Foz do Iguaçu, PR, Brazil.

**Keywords:** Covid-19, Child Care, Premature, Child Development, Health Promotion, Telemedicine, Covid-19, Cuidado da Criança, Prematuro, Desenvolvimento Infantil, Promoção da Saúde, Telemedicina, Covid-19, Cuidado del Niño, Prematuro, Desarrollo Infantil, Promoción de la Salud, Telemedicina

## Abstract

**Objective:**

to analyze elements of the follow-up care provided to premature children amidst the COVID-19 pandemic.

**Method:**

qualitative study from the perspective of philosophical hermeneutics, interpreting experiences with childcare provided at home. Twelve mothers and 14 children aged two years old were interviewed online via a text messaging application. Data were analyzed by interpreting meanings.

**Results:**

weaknesses stood out in the follow-up care provided to children such as gaps of communication, lack of guidance and delayed immunizations, while care intended to meet health demands was interrupted. Vulnerability aspects affecting child development included: social isolation measures that impeded the children from socializing with their peers, increased screen time, the manifestation of demanding behaviors and irritation and the mothers experiencing an overload of responsibilities. The elements that strengthened maternal care included the mothers being attentive to contagion, enjoying greater experience and satisfaction with the maternal role, spending more time with their children, and recognizing respiratory signs and symptoms, especially fever.

**Conclusion:**

follow-up care provided to children in stressful situations implies implementing practices that support the wellbeing of children and families, decreasing the likelihood of children being exposed to development deficits, and detecting signs and symptoms timely. The use of nursing call centers can break the invisibility of longitudinal needs and promote health education actions at home.

## Abstract (pt)

**Objetivo:**

analisar os elementos relacionados ao seguimento da saúde da criança com histórico de prematuridade em meio à pandemia da COVID-19.

**Método:**

estudo qualitativo na perspectiva da hermenêutica filosófica, conduzido pelo movimento interpretativo das experiências de cuidado da criança em casa. Participaram 12 mães e 14 crianças de dois anos de idade, com entrevistas *on-line* por mensagem de texto instantânea e análise de dados por interpretação de sentidos.

**Resultados:**

destacaram-se elementos frágeis ao seguimento da saúde da criança: lacunas na comunicação, ausência de orientações, atraso vacinal, demandas de cuidados interrompidas; elementos vulneráveis ao desenvolvimento infantil: distanciamento social impeditivo ao convívio entre pares, aumento do uso de telas, comportamentos de irritação e reivindicações, sobrecarga das atribuições maternas; e elementos fortalecedores dos cuidados maternos: atenção ao contágio, experiência e satisfação no papel materno, ampliação do tempo de convívio com a criança, reconhecimento de sinais e sintomas respiratórios, principalmente febris.

**Conclusão:**

o seguimento da saúde das crianças em situações estressoras implica ampliar práticas sustentadoras ao bem-estar infantil e familiar, reduzir chances de expor crianças aos prejuízos no desenvolvimento e detectar oportunamente sinais e sintomas. O teleatendimento de enfermagem pode quebrar a invisibilidade das necessidades longitudinais e alavancar as ações de educação em saúde em domicílio.

## Abstract (es)

**Objetivo:**

analizar elementos del seguimiento de la salud del niño con historial de prematuridad en medio de la pandemia de la COVID-19.

**Método:**

estudio cualitativo en la perspectiva de la hermenéutica filosófica, conducido por el movimiento interpretativo de las experiencias de cuidado del niño en el hogar. Participaron 12 madres y 14 niños de 2 años de edad, con entrevistas *online* por mensaje de texto instantáneo y análisis de datos por interpretación de sentidos.

**Resultados:**

se destacaron elementos frágiles al seguimiento de la salud del niño: lagunas en la comunicación, ausencia de orientaciones, atraso de vacunación, demandas de cuidados interrumpidas; elementos vulnerables al desarrollo infantil: distanciamiento social impeditivo a la convivencia entre pares, aumento del uso de pantallas, comportamientos de irritación y reivindicaciones, sobrecarga de las atribuciones maternas; y elementos fortalecedores de los cuidados maternos: atención al contagio, experiencia y satisfacción en el papel materno, ampliación del tiempo de convivencia con el niño, reconocimiento de señales y síntomas respiratorios, principalmente febriles.

**Conclusión:**

el seguimiento de la salud de los niños en situaciones estresantes implica ampliar prácticas sustentadoras al bienestar infantil y familiar, reducir posibilidades de exponer niños a los perjuicios en el desarrollo y detectar oportunamente señales y síntomas. Los servicios remotos de enfermería pueden quebrar la invisibilidad de las necesidades longitudinales y apalancar las acciones de educación en salud a domicilio.

## Introduction

The disease named COVID-19 is caused by the Coronavirus-2 of the Severe Acute Respiratory Syndrome (SARS-CoV-2), an acute and rapidly evolving respiratory disease, first described in Wuhan, China, in December 2019^(^ 1 ^)^. Its primary signs and symptoms described thus far include dyspnea, fever, dry cough, body ache, sore throat, runny nose, vomiting, diarrhea, and skin rashes, while severe cases lead to progressive respiratory failure and require immediate ventilatory support^(^ 2 ^-^ 3 ^)^.

Many people in various countries have been infected by this disease in 2020 and mortality rates range from 0.5% to 18% according to age; a greater incidence is verified among individuals aged 60+ years old and with comorbidities^(^ 4 ^)^. Susceptibility among individuals younger than 20 years of age is approximately half that among individuals over 20 years of age, suggesting that SARS-CoV-2 is twice less likely to infect children and adolescents up to the age of 19, and most of those who become infected in this age group are asymptomatic^(^ 5 ^)^.

Relatively few children under 10 years old have been diagnosed with COVID-19, representing from 1% to 5% of infected cases^(^ 6 ^)^. Additionally, physical symptoms have been less aggressive and hospitalizations less frequent in this age group^(^ 6 ^-^ 7 ^)^. Nonetheless, even though the disease seems to be less severe among children, severe cases that require hospitalization are still a possibility and may progress to death, especially among children with chronic health problems^(^ 6 ^-^ 7 ^)^.

Among the group of children with chronic health problems are those born prematurely, who generally experienced lengthy hospitalizations after birth, with adverse conditions accruing from a variety of disorders and interventions, which results in a greater likelihood of disabilities, infections, complications, re-hospitalizations, and changes in growth and development in the short or long terms^(^ 8 ^-^ 10 ^)^. Thus, monitoring these children’s health is vital, and even though the occurrence of COVID-19 tends to be less frequent among children, assistance to the needs of these children may be hindered due to the pandemic, considering the social isolation measures imposed to control the spread of the coronavirus^(^ 11 ^)^.

In pandemic situations, institutional health weaknesses may become more pronounced, considering the many emerging demands requiring attention, so that health promotion and disease prevention actions end up in the background amidst the potential collapse of the health system^(^ 12 ^)^. On the other hand, the follow-up care provided to premature children needs to be structured^(^ 9 ^)^, including interventions to deal with adversities^(^ 13 ^)^ and avoid fragilities that may affect parents^(^ 14 ^)^, causing problems that prevents integral health care. In this sense, this study assumes that the parental practices of mothers of children who were born prematurely have additional needs to monitor the health of their children when facing stressful situations. Hence, this study’s objective is to analyze the elements concerning the follow-up care provided to children who were born prematurely amidst the COVID-19 pandemic.

## Method

This qualitative study was conducted from the perspective of the philosophical hermeneutic approach^(^ 15 ^)^, based on a dialogical interpretation of maternal experiences with childcare needs.

This study was conducted in Foz do Iguaçu, PR, Brazil, a city that belongs to the triple border near Ciudad del Este in Paraguay and Puerto Iguazu in Argentina. There is only one Neonatal Intensive Therapy Unit in this city, with care provided to patients covered by the public and private systems. It has high technological density and is the only facility responsible for providing care to high-risk newborns from the cities covered by the Foz de Iguaçu health region, to the newborns of tourists visiting the city, and internationals living in the countries in the triple border. There is also a Neonatal Intermediate Care Unit and a Kangaroo Unit in the same hospital facility, though mothers are not allowed to spend the night with their children in these units. Follow-up care of patients covered by the private system is provided by private pediatrician offices.

The follow-up of premature infants covered by the public health system is performed by the Child Nutrition Center after they are discharged from the hospital until the age of 14 years old. At this service, physicians, nurses, and nutritionists monitor the health of premature children and low birth weight. Childcare outpatient services were temporally suspended at the beginning of the COVID-19 pandemic and were closed at the time of data collection.

The sample was composed of mothers aged ≥18 years old, with a gestational age below 37 weeks at the time of delivery, whose children were hospitalized immediately after birth, living in Foz do Iguaçu, PR, Brazil. Mothers with a diagnosis of mental disorders recorded on their medical files, with communication problems due to the ethnic diversity of the triple border region, or who were not encountered due to change of telephone number or address, were excluded.

The intentional sample was formed by 12 mothers with 14 children (from the twins) with a history of prematurity, who answered the telephone contacts of the researcher.

Data were collected using a semi-structured form, addressing aspects that concerned the follow-up care of premature children amidst the COVID-19 pandemic. This study’s guiding question was: “Could you please tell me how you have been taking care and monitoring the health of your premature child amidst the COVID-19 pandemic?”. Other questions composed the form: whether the children presented special health needs, using the screening instrument CSHCN *-* Children with Special Healthcare Needs^(^ 16 ^)^; maternal concerns with COVID-19 and social distancing measures; immunizations; how the mothers were handling fevers and flu symptoms; activities/games at home; orientations provided by health workers; and maternal feelings concerning the care provided to their children at home.

This study’s primary author, experienced in neonatal nursing, collected data between May and June 2020. The technique used was online interviews, conducted via a text messaging application (WhatsApp). The guiding question was asked first, allowing the participants to express their experiences, and then the remaining questions followed. The participants recorded audio (five contacts with audio messages that lasted between 5 and 10 minutes) and texted messages (22 contacts, between 3 and 25 messages). Four participants exchanged messages for three days and the messages of eight participants were distributed in two days. The text and audio messages were later transcribed verbatim and made available online for the participants to approve their content.

Data analysis included reading the empirical material more than once to interpret the meanings, which enabled a broad view of the whole in line with the analysis of its particularities, focusing on experiences that concerned the follow-up care provided to the children amidst the COVID-19 pandemic, seeking to understand and explore meanings^(^ 17 ^)^. Therefore, data analysis consisted of a comprehensive reading of the material, developing a structure of analysis, and searching for broader meanings^(^ 17 ^)^.

The context of the mother’s experiences at home and in the follow-up care provided to the children amidst the COVID-19 pandemic enabled thematic units to emerge, namely: The COVID-19 pandemic and children’s health: maternal concerns and attention paid to contagion; Social distancing and its repercussions on the children’s development; Gaps and challenges faced in the follow-up care of premature children amidst the COVID-19 pandemic.

The study was approved by the Institutional Review Board following guidelines for research involving human subjects (Opinion report 4.051.005). To ensure that the participants’ identities remained confidential, they were identified with the letter P followed by a number (P1 to P12).

## Results

The premature children were born with a gestational age between 30 and 35 weeks and remained hospitalized from 5 to 58 days after birth. The children’s age ranged from 2 years and 7 months to 2 years and 10 months. None of the children had their health compromised at the time of the online interviews and the members of only one family presented COVID-19 symptoms, though the diagnosis was not confirmed. Eight of the 14 children (two twins) were attending follow-up visits at Primary Health Care (PHC) services and six were attending follow-up visits in private services; the immunization of 12 children was updated, and two had not been vaccinated for influenza; one child had been hospitalized due to respiratory symptoms before the beginning of the pandemic.

*The COVID-19 pandemic and children’s health: maternal concerns and attention paid to contagion*: The mothers expressed their fears concerning the health and protection of their children and remaining family members since the emergence of the COVID-19, which involved paying attention to the potential risk of contagion. *The concern right now is to stay healthy, take care of ourselves so nobody gets sick at home* (P1); *I’m concerned with the mass contagion with COVID-19, increasing the likelihood of us getting contaminated* (P4); *I’m afraid my family and friends get contaminated* (P8).

Because the mothers were concerned with the health of their children and families, they reported important actions to prevent contamination by the coronavirus. *Wearing masks, washing hands, using alcohol, so nobody gets sick. We are complying with isolation measures; we only leave the house to buy the essential, not taking any chances* (P1); *We are taking all precautions with hygiene, staying at home, and taking preventive measures* (P9); *I’m really afraid, for this reason, we’re not leaving the home, always wearing masks and washing hands* (P10).

Prematurity was seen by the mothers as an aspect that can weaken the health of their children in the context of the COVID-19 pandemic. *I’m concerned with my little ones* [twins]*. Every premature child is sensitive, I don’t know, I’m afraid because the family comes to visit, I’m afraid they are contaminated* (P3); *I’m concerned because she gets sick due to any silly thing. If the weather gets a bit chilly, if we don’t take care, she gets sick. I’m concerned and very afraid because of her. You know, any little thing, she’s the weakest in the house* (P6); *Because she’s premature, I’m afraid she won’t resist in case she catches the virus* (P12).

The mothers reported that family life configured a concern due to fear that their children, who they considered to be fragile, would be contaminated: *I’m concerned because my husband smokes near to her, we tell him, but he won’t listen* (P6); *I’m concerned with getting the virus or someone in the family, from going to the grocery store, public places. Of course, I don’t take him with me, but I’m actually worried about everything* (P5); *My main concern is that someone gets it and transmit it to my baby, we don’t know how the disease behaves in children, especially babies. They say children have mild symptoms, but there is no certainty* (P11).

Additionally, the mothers reported concerns related to other health problems their children may experience, considering their fragility due to the premature birth. They do not receive the assistance they need though, considering that the follow-up services were suspended due to the pandemic. *My greatest concern is with his health, you know? If he gets sick, with some problem other than the COVID-19. I’m worried that he won’t get assistance because the public health system is already bad, now is even worse. They are focusing so much on COVID-19 that I believe other diseases are being disregarded* (P5).

*Social distancing and its repercussions on the children’s development:* This measure was perceived by some mothers as something that can interfere in the development of their premature children. *This period at home, not being able to go to daycare, not learning anything at daycare. We do some activities at home so she won’t be idle* (P1); *Children need to socialize to develop* (P2); *I got a little concerned with their* [twins] *development because I believe they need to socialize with other children to learn more* (P3).

Some mothers reported a concern with the need to meet the development needs of their premature children, which were interrupted by the COVID-19 pandemic. *I’m concerned with her development because treatments such as physical therapy were interrupted, as well as other therapies* (P4).

The mothers reported difficulties to perform their day-to-day tasks with their children at home. *It’s difficult because her only distraction is to watch cartoons on TV, she doesn’t have contact with other children* (P1); *She’s been very irritated, I need to buy games like the ones they have in the daycare. Everything is fine, we make do, always play with her when we can* (P6); *We’d go outside every day. So, this is difficult, having to stay home, but now we are getting used to the new routine. We’re playing a lot and the mobile is helping a lot with child movies. So, I can do some household chores* (P9).

On the other hand, the mothers believe this pandemic will not last long. So some mothers believe this social distancing period will not harm the future of their children, especially because they provide stimuli at home in their daily routine. *I believe she is losing content, not anything that will impact the rest of her life, but content that she will catch up with* (P1)*; I’m not concerned with his development because I believe this will pass. God, I hope it will pass soon. I’m trying to stimulate him as well as I can at home, but of course, it is not the same as socializing with other children* (P5); *I was not concerned because I always taught my daughters at home and she is way ahead of her peers; the teachers say it themselves* (P9).

Amidst social distancing, the mothers report that they have established a routine to deal with the difficulties faced in the care of their children. *I like to tell them biblical stores, I’m Evangelic. I also like to tell them their* [twins] *history, that one day a couple had two tiny little babies, who needed to stay at the hospital* (P3); *I’m reading books she loves, things I download on my mobile. We watch a little TV because there is so much to do that there isn’t much time to watch TV with her. She spends some time in the afternoon with the mobile. We play at home, play ball, which she loves, and we make up new games such as teaching her to ride her tricycle, bicycle, run with the dog* (P9).

The social distancing measures also elicited aspects related to life with the child, playing the maternal role while experiencing feelings of uncertainty. *There is the financial situation, which does not help. If I could I’d send him to daycare only later, I’d care for him most of the time if I could, When I’m with him, I try to spend as much time as I can and give him as much attention as I can because he’s in a phase he needs me* (P5); *I’m very happy about being a mom and about how I’m doing* (P8); *I agree that my children* [twins] *demand a lot of my energy, but all my time is for them* (P2).

To care for small children with dedication and patience has not been an easy task for these mothers, considering the stressful situation in the context of the COVID-19 pandemic. *Everything in life that is worthwhile demands work and children are a handful, we know. As a mother, sometimes, I get crazy with his tantrums. There’s always something they want and you can’t give them. But, I don’t know, after everything I’ve been through, the fact that he was born prematurely, I’m more patient than when I didn’t have a child. It is tiresome, but it isn’t frustrating for me. I like it, and I don’t see my life without him* (P5); *You notice she is screaming in the back because now is her time with the mobile and I’m using it. So, she got really mad* (P9); *I like to spend time with my child* (P10).

The care provided to the children resulted in the mothers having little time for themselves, making their lives hardly flexible and often overburdened. *My children* [twins] *leave little time to take care of myself* (P3); *Dear, there’s no time for anything with them at home* (P6).

The mothers acknowledged that social distancing measures have affected their daily lives, especially their children’s development. The children became irritated, tired, and demanded the mothers’ attention and interaction but the mothers have been able to alleviate this critical time the families are facing.

*Gaps and challenges faced in the follow-up care of premature children amidst the COVID-19 pandemic:* With the emergence of SARS-CoV-2, the mothers found out that the follow-up care provided to the children in the PHC services had been temporarily discontinued. *Up to one year old, I used to take them to the Child Nutrition Center, but then I stopped because it was difficult for me, too far away from home, and with two children* [twins]*. After that, I started taking them to the health unit, but not anymore* (P3); *I didn’t take him to get the flu vaccine. I forgot to ask about the vaccine when I called the health unit, but I’ll call them on Monday* (P5); *Her last follow-up visit was in January, at the Nutrition Center, where she attends follow-up since she was discharged from the hospital. Now I don’t know when I’ll take her there again* (P9); *I’m waiting to give her the flu vaccine, but all the others are up to date* (P1).

In addition to the COVID-19 pandemic, the mothers reported family problems as an aspect that hinders the follow-up of their children. *My husband and I fought, I got beaten up and have lost a baby. We lived apart for six months. I was alone, living by myself, having to take care of everything. After that, I stopped taking the baby to all the follow-up visits* (P6).

In addition to personal difficulties to maintain the follow-up visits, the mothers also reported that the support provided by the health workers amidst the COVID-19 pandemic was poor and had been interrupted. *The health unit did not provide any guidance, didn’t even call* (P2); *The health unit didn’t call during the distancing measures. Actually, I called them to know whether the unit would be working in case I needed to take my child there, but they didn’t call, not even once* (P5); *Nobody called me from the unit to say anything about COVID, how we should take care of ourselves. I’m taking care of myself, washing hands frequently* (P6).

Regarding fever, a symptom most individuals infected with COVID-19 present, the mothers reported measures to manage it, including measuring the children’s temperature, bathing them, and seeking the health services in case the symptoms persisted. *If I see that the fever is not going away, I first do two things: give the child a bath and medication. If I see it is persistent, then I take the child to the doctor. I have a thermometer at home and know how to use it* (P1); *If he has a temperature, I give him a bath and medication, I won’t stay at home with a sick child, I won’t. I freak out a bit, I get worried, I’m afraid of giving the wrong medication* (P5); *If she has a fever, I give her medication. I keep tabs of her temperature, I have a thermometer and know how to measure it. If she has a fever for three days, I take her to the doctor* (P12).

Likewise, in the presence of flu symptoms, the mothers reported understanding and the challenges of taking care of children. *If she is coughing, I give her cough syrup, but if it takes more than one day, the cough is not getting better, I take her to the doctor. If she has a runny nose, I usually wait one or two days, if she doesn’t get better, I take her to the doctor* (P1); *If she coughs, I use medication, some syrup, I make teas, and take her to the emergency room as a last resort* (P4); *In case of cough, I prepare some tea and take the child to the doctor. In case of a runny nose, I use a saline spray and take the child to the doctor* (P10).

In this study, among the children who were classified as having special health needs (CSHCN), one child had brain damage and requires specialized monitoring, and another has a chronic respiratory problem and, for this reason, takes medications regularly. *My daughter has a mental problem, she needs physical therapy, hippotherapy, occupational therapy, hydrotherapy, and visual stimulation. I don’t know for how long she’ll need these* (P4); *My daughter has a respiratory problem. She received respiratory physical therapy for a while, but not anymore. She takes medication such as Aerolin, Seretide, Avamys, and Montelair. I don’t know for how long she will take these* (P12).

The mothers reported their children’s care needs and the challenges faced to meet these needs while there are no perspectives of when the service will resume.

**Figure 1:**
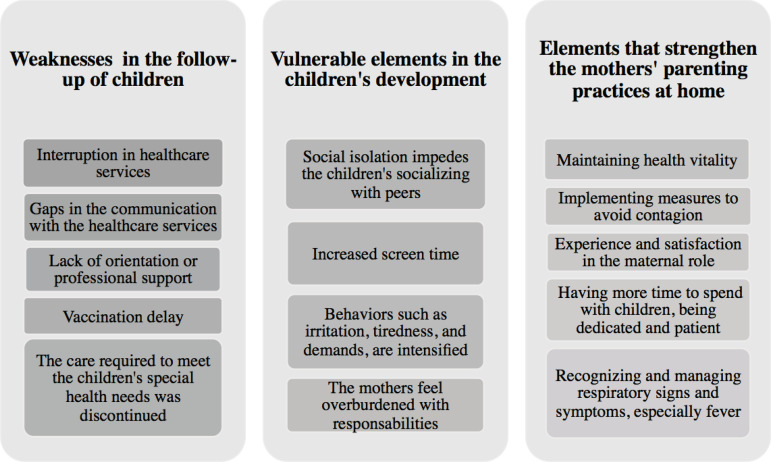
Vulnerable and protective elements in the follow-up care and home care of premature children amidst the COVID-19 pandemic. Foz do Iguaçu, PR, Brazil, 2020

Figure 1 presents the qualitative elements that emerged from the online interviews, including weaknesses and strengths regarding follow-up care and care provided at home to premature children.

## Discussion

This study presents situational elements and the repercussions of the COVID-19 pandemic for premature children. This context and the situations emerging from it reveal vulnerabilities, which from the mothers’ perspective may make their children more susceptible to the COVID-19. The fact that the follow-up care was interrupted revealed a disconnection with the children’s needs and the care provided at home.

In the context of parental practices, the mothers report care actions and measures they implement to take care of their children at home, however, the children’s fragility because they were born prematurely, more strongly emphasized within the pandemic context, includes doubts regarding the follow-up visits. Vulnerable elements in the children’s development suggest the need to improve professional support to protect and watch for the health of children and families.

Prematurity is a factor that predicts that the mothers will be committed to the care provided at home, with implications that may affect mother-baby attachment and child development^(^ 18 ^)^. When parental practices are affected by concerns such as the COVID-19 pandemic, caregivers, especially mothers, may become more susceptible to stressful factors, with a strong connection between perceived and physiological stress^(^ 19 ^)^. At these times, interactions are affected and interventions need to be implemented at home to strengthen the motivation and the relationship within the families^(^ 18 ^)^, considering that changes in the family and social context lead to an overload of responsibilities that weaken the care provided to the child, home and family^(^ 20 ^)^.

The mothers reported practices intended to promote the development of their children, however, using screen time to keep the children busy, whenever they are stressed or throw a tantrum, or to allow the mothers to do household chores, may be dysfunctional in developmental terms. The electronic media is seen as a tool to support caregivers, allowing them more time to perform household tasks or take care of themselves, or just to keep the children busy and entertained^(^ 21 ^-^ 22 ^)^. The literature shows the need to provide guidance though, explaining in detail how the exposure of children to various electronic devices and increased screen time is a source of concern^(^ 23 ^)^.

The mothers consider that the effects of social distancing on the children’s development are permeated by the notion that the pandemic is temporary. They consider the fact the children cannot socialize with their peers as negative but reported positive prospects for the children’s development. These results suggest that they are capable to face adversities and adapt to the current context, though sometimes they are realistic and sometimes optimistic.

Countless socioenvironmental factors may influence the development of two-year-old premature children. Being deprived of playing and participating in group activities may lead to complications in the short and long term^(^ 24 ^)^. In these circumstances, empathy and child-parent interactions are vital to promoting child development, such as watching a cartoon together, reading books, and playing together, adapting to the peculiar needs of each child, according to each child’s rhythm, times, and situations^(^ 25 ^)^.

When facing stressful experiences, even families concerned with providing care and *stimuli* at home to their children and keeping up positive thinking that everything will pass, there will be times in which the family will be preoccupied with other concerns, such as the economic impact caused by the pandemic, considering the need to provide for the family’s subsistence and to provide additional care the children may require. Note that the Foz do Iguaçu’s main economic activity is tourism, which directly or indirectly contributes to the families’ income^(^ 26 ^)^, and the tourism sector was severely affected by the pandemic worldwide. The perception of child development may become circumstantial, with the possibility of increasing parental stress, negatively reflecting on care, and increasing intra-family vulnerability^(^ 27 ^)^.

Regarding the children’s follow-up care, the mothers’ reports reveal weaknesses such as the abrupt interruption of health care services and monitoring, suggesting that performing an active search through home visits and/or providing support via telephone are important tools that can be used in the health field, but which the PHC service did not adopt.

The support provided by health workers to families with premature children is essential to promote health and prevent diseases^(^ 28 ^-^ 29 ^)^, as well as to reinforce measures to control the spread of COVID-19^(^ 5 ^,^ 7 ^)^. A study conducted in the same context addressed here reports that there are important vulnerabilities in the continuity of care provided to premature children, showing a deep disconnection between PHC, hospital-based and specialized services^(^ 28 ^)^. Gaps in the follow-up care of premature children with lengthy hospitalizations are a source of concern in the face of the pandemic, which make it even more challenging to promote health and prevent diseases, considering the previously existing difficulties faced in the public health system, now overburdened, hindering the care provided to children.

Additionally, the vulnerability of children due to prematurity, linked to morbidities and mortality, complications, and iatrogenic problems from the time of hospitalization^(^ 28 ^)^, may lead children with special health needs to experience new care needs^(^ 30 ^)^. The mothers report that the need of their children for healthcare assistance in the pandemic context and the health services’ inability to provide follow-up care is very apparent in the current context.

The mothers recognize the vulnerability of their children due to the fact they were born prematurely and are vigilant for signs of complications already identified in another study^(^ 10 ^)^, which are similar to the reports concerning flu symptoms and fever. Regarding COVID-19 symptoms, the participants are attentive and concerned with the risk of contagion. Even though the number of children infected with COVID-19 has been small thus far, children are vulnerable to the infection^(^ 7 ^)^, which justifies the need to improve guidance and reinforce control measures.

The COVID-19 pandemic is a severe disease considered a public health emergency worldwide. It has led health facilities to reorganize and redirect services and transformed the routine of families, with severe implications for human development and behavior. This context is permeated by fear, uncertainties, and challenges, indicating that the families need to receive support to promote parental care and keep positive attitudes with their children at home^(^ 31 ^)^.

In this sense, the social isolation measures may be seen as a promising opportunity for the families to construct sustainable relationships, to reserve time to interact with their children, find out what their children like, and do things together. It is also a valuable opportunity to dialogue, be affectionate, and show mutual importance, promoting good child development^(^ 31 ^)^. Therefore, interventions during the children’s first years of life are essential, benefiting integral health^(^ 32 ^)^, minimizing vulnerabilities, and expanding inter-subjective experiences, based on the notion that experiences include interactions and attention is paid to new and different possibilities^(^ 15 ^)^.

Note that diseases with complex transmission patterns, including environmental, social, economic, or unknown determinants, are considered difficult to control^(^ 33 ^)^, with implications for long-term treatment and preventive measures, demanding the same surveillance required by communicable diseases^(^ 34 ^)^.

It is worth noting that the Brazilian Ministry of Health suggested, within the pandemic context, to temporarily suspend elective appointments for premature asymptomatic babies in the hospitals’ outpatient follow-up clinic^(^ 35 ^)^. The recommendation was to keep elective follow-up visits in the PHC services, considering that these visits are an opportunity to implement routine immunization, monitor growth and development, and provide guidance to the families^(^ 35 ^)^.

The work performed by nurses in PHC services is essential in the care provided to premature children within a pandemic context. The reason is that nurses can manage care and develop actions together with the remaining members of the staff, sectors, and services, intending to encourage the follow-up care of premature children and strengthen parental practices at home, identifying the elements highlighted in this study that protect the children or make them vulnerable.

These results contribute to health practices and encourage health workers to support caregivers in vulnerable times, using strategies such as telenursing, which can promote wellbeing and decrease the risks to which children are exposed when experiencing adverse situations. Telenursing can break the invisibility of longitudinal needs, facilitate health education, and promote adherence to protective measures, important to the follow-up care of children, including those with special health needs, and to strengthen good parenting practices.

This study’s limitations involve the fact the interviews were held online, which may have restricted the perception of characteristics inherent to deeper dimensions of the phenomena under analysis. This investigation reveals qualitative elements of the online interviews that facilitate an understanding of singular experiences at a time when social distancing measures were imposed to control the spread of a severe disease.

## Conclusion

Peculiarities of the domestic environment and uncertainties concerning the repercussions of COVID-19 for children, especially considering the fragilities and possibility of presenting other health problems, coupled with preexisting difficulties presented by the health system, were factors that caused the families to feel insecure and unprotected.

The nursing practice within PHC gains a new focus, which comes with a recognition that this pandemic and consequent social distancing measures interfere with the lives of children and their families. Gaps concerning health actions in vulnerable situations such as among premature children and the current pandemic, magnify weaknesses in the follow-up care and monitoring of children.

The work of nurses can contribute to establishing a bridge with parental caregivers to improve their ability to respond to adversities and challenges healthily, promote learning on how to provide care in situations in which emotions are contradictory, and increment inter-subjective experiences, which are favored by the bond established with families. Thus, nurses promote an environment where sustainable care actions preserve the children’s and families’ wellbeing, reducing the likelihood of exposing children to developmental deficits, refraining the dissemination of the virus by efficiently decreasing the risks of contamination, detecting signs and symptoms timely, preventing adverse experiences and increasing the population’s trust in scientifically grounded health guidance.

## References

[1] Wenzhong L, Hualan L (2020). Covid-19: attacks the 1-Beta chain of hemoglobin and captures the porphyrin to inhibit human heme metabolism. Chem Rxiv.

[2] Xu Z, Shi L, Wang Y, Zhang J, Huang L, Zhang C (2020). Pathological findings of Covid-19 associated with acute respiratory distress syndrome. Lancet Respir Med.

[3] Joob B, Wiwanitkit V (2020). Covid-19 can present with a rash and be mistaken for dengue. J Am Acad Dermatol.

[4] World Health Organization (2020). Coronavirus (COVID-19).

[5] Davies NG, Klepac P, Liu Y, Prem K, Jit M, CMMID Covid-19 working group (2020). Age-dependent effects in the transmission and control of Covid-19 epidemics. Nat Med.

[6] Ludvigsson JF (2020). Systematic review of Covid-19 in children shows milder cases and a better prognosis than adults. Acta Paediatr.

[7] Vilelas JMS (2020). The new coronavirus and the risk to children's health. Rev. Latino-Am. Enfermagem.

[8] Rover MMS, Viera CS, Toso BRGO, Grassiolli S, Bugs BM (2015). Growth of very low birth weight preterm until 12 months of corrected age. J Hum Growth Dev.

[9] Voller SMB (2018). Follow-up care for high-risk preterm infants. Pediatr Ann.

[10] Steiner L, Diesner SC, Voitl P (2019). Risk of infection in the first year of life in preterm children: an Austrian observational study. PLoS One.

[11] Nussbaumer-Streit B, Mayr V, Dobrescu AL, Chapman A, Persad E, Klerings I (2020). Quarantine alone or in combination with order public health measures to control Covid-19: a rapid review. Cochrane Database Syst Rev.

[12] Litewka SG, Heitman E (2020). Latin American healthcare systems in times of pandemic. Dev World Bioeth.

[13] McGowan EC, Vohr BR (2019). Neurodevelopmental follow-up of preterm infants: what is new?. Pediatr Clin North Am.

[14] Cprek SE, Williams CM, Asaolu I, Alexander LA, Vanderpool RC (2015). Three positive parenting practices and their correlation with risk of childhood developmental, social, or behavioral delays: an analysis of the National Survey of Children's Health. Matern Child Health J.

[15] Gadamer H (2014). Verdade e método: traços fundamentais de uma hermenêutica filosófica.

[16] Arrué AM, Neves ET, Magnano TSBS, Cabral IE, Gama SGN, Hökerberg YHM (2016). Tradução e adaptação do Children with Special Health Care Needs Screener para português do Brasil. Cad Saude Publica.

[17] Gomes R, Minayo MCS (2010). Análise e interpretação de dados de pesquisa qualitativa. Pesquisa social: teoria, método e criatividade.

[18] Anderson C, Cacola P (2017). Implications of preterm birth for maternal mental health and infant development. Am J Matern Child.

[19] Garfield CF, Simon C, Rutsohn J, Lee YS (2018). Stress from the neonatal intensive care unit to home - paternal and maternal cortisol rhythms in parents of premature infants. J Perinat Neonatal Nurs.

[20] Granero-Molina J, Medina IMF, Fernández-Sola C, Hernández-Padilla JM, Lasserrotte MMJ, Rodríguez MML (2019). Experiences of mothers of extremely preterm infants after hospital discharge. J Ped Nurs.

[21] Vittrup B, Snider S, Rose KK, Rippy J (2016). Parental perceptions of the role of media and technology in their young children's lives. J Early Child Res.

[22] Pempek TA, McDaniel BT (2016). Young children's tablet use and associations with maternal well-being. J Child Fam Stud.

[23] Chang HY, Park E, Yoo H, Lee JW, Shin Y (2018). Electronic media exposure and use among toddlers. Psychiatry Investig.

[24] Kenyhercz F, Nagy B (2017). Examination of psychomotor development in relation to social-environmental factors in preterm children at 2 years old. Orv Hetil.

[25] Brazelton TB, Greenspan SI (2002). As necessidades essenciais das crianças: o que toda criança precisa para crescer, aprender e se desenvolver.

[26] Aikes S, Rizzotto MLF (2018). Regional integration of healthcare services in twin cities, Paraná State, Brazil. Cad Saude Publica.

[27] Cluver L, Lachman JM, Sherr L, Wessels I, Krug E, Rakotomalala S (2020). Parenting in a time of Covid-19. Lancet.

[28] Berres R, Baggio MA (2020). (Dis)continuation of care of the pre-term newborn at the border. Rev Bras Enferm.

[29] Casey PH, Irby C, Withers S, Dorsey S, Li J, Rettiganti M (2017). Home visiting and the health of preterm infants. Clin Pediatr.

[30] Pieszak GM, Neves ET (2020). Family care for children with special health needs and social care networks. Res Soc Dev.

[31] Fundação Maria Cecília Souto Vidigal (2020). Covid-19: cuidados parentais.

[32] Venancio SI (2020). Why invest in early childhood. Rev. Latino-Am. Enfermagem.

[33] Barreto ML, Teixeira MG, Bastos FI, Ximenes RA, Barata RB, Rodrigues LC (2011). Successes and failures in the control of infectious in Brazil: social and environmental context, policies, interventions, and research needs. Lancet.

[34] Ameli J (2016). Communicable diseases and outbreak control. Turk J Emerg Med.

[35] Ministério da Saúde (BR) (2020). Nota Técnica nº6/2020. Atenção à saúde do recém-nascido no contexto da infecção pelo novo coronavirus.

